# A Practical Approach to Systemic Mastocytosis Complications in Cardiac Surgery: A Case Report and Systematic Review of the Literature

**DOI:** 10.3390/jcm12031156

**Published:** 2023-02-01

**Authors:** Mathieu N. Suleiman, Valeska Brueckl, Jörg Fechner, Ann-Sophie Kaemmerer, Florian Wilk, Michael Weyand, Frank Harig

**Affiliations:** 1 Department of Cardiac Surgery, University Hospital Erlangen, Friedrich-Alexander-University Erlangen-Nürnberg, 91054 Erlangen, Germany; 2 Department of Haematology and Oncology, University Hospital Erlangen, Friedrich-Alexander-University Erlangen-Nürnberg, 91054 Erlangen, Germany; 3 Department of Anesthesiology, University Hospital Erlangen, Friedrich-Alexander-University Erlangen-Nürnberg, 91054 Erlangen, Germany

**Keywords:** systemic mastocytosis, shock, anaphylaxis, protamine, cardiac surgery, cardiac anesthesia

## Abstract

(1) Background: Systemic mastocytosis is a rare, non-curable disease with potential life-threatening complications in patients receiving cardiac surgery. (2) Methods: This systematic review of the literature was prompted by the case of a life-threatening anaphylactic reaction during cardiac surgery related to systemic mastocytosis. The search of all types of studies, using several databases (Pubmed, Scopus and Web of Science), was conducted through September 2022 to identify the relevant studies. (3) Results: Twelve studies were included describing cases of patients undergoing cardiac surgery who were diagnosed with systemic mastocytosis. An adverse effect, namely anaphylaxis, has happened in three cases. Different strategies of premedication, intraoperative and postoperative management were used. In our case, the patient was admitted for elective biological aortic valve replacement due to severe aortic stenosis. Intraoperatively, the patient developed an anaphylactic shock during the administration of protamine after separation from the cardiopulmonary bypass. This anaphylaxis reaction was a complication of the pre-existing systemic mastocytosis and could be successfully managed by the administration of epinephrine, antihistamines and corticosteroids. (4) Conclusions: This systematic literature search and case report highlight the importance of careful preoperative planning, as well as coordination between cardiac surgeons, anesthesiologists and hemato-oncological specialists, in patients with rare but complication-prone diseases such as systemic mastocytosis.

## 1. Introduction

Systemic mastocytosis (SM) is a rare disease in which mast cells accumulate under the skin and/or tissues, especially the bone marrow, putting patients at high risk of dangerous anaphylactic reactions from mast cell degranulation [1].

Most patients with SM have activating, function-enhancing mutations in a transmembrane tyrosine kinase receptor for stem cell factor (c-KIT) in their neoplastic mast cells. Caused by the mutation in the KIT gene (codon 816 mutation), patients with SM develop an abnormal proliferation of their neoplastic mast cells, a type of white blood cell and part of the immune system found in connective tissue [2].

SM clinically manifests in different forms depending on which organs and tissues are affected. Usually, neoplastic mast cells accumulate in various tissues and organs, including the liver, spleen, bone marrow, small intestine, skin (urticaria pigmentosa) and lymph nodes [3]. The degranulation of mast cells, if triggered by chemical, physical or psychological factors, can cause an allergic reaction, anaphylactic shock and severe systemic inflammation with significant cardiovascular compromise and subsequent organ damage [4,5,6]. 

Survival rates depend on the type and severity of mastocytosis. While life expectancy is almost normal in indolent mastocytosis, aggressive forms of mastocytosis, namely advanced systemic mastocytosis (ASM), may have a prognosis of only a few years after diagnosis. 

Although cardiac surgery with cardiopulmonary bypass (CPB) may provoke the uninhibited release of mast cell mediators and severe allergic reactions, even today only limited data are available on the optimal perioperative management of patients with SM undergoing cardiac surgery [4]. Therefore, it is essential to avoid or prevent all trigger factors, if possible. 

As SM is a rare disease, the information on the perioperative management is, at present, only sparsely available in the literature through case reports. Thus, no guidelines are present to help ease preoperative planning in patients with SM receiving cardiac surgery. Thus, the results of our study could be helpful to facilitate decisions between the cardiac surgeon, anesthesiologist and hemato-oncologist and could provide some guidance in the management of these patients.

## 2. Case Presentation

A 66-year-old woman (164 cm, 78.8 kg, BMI: 29.3 kg/m^2^), diagnosed with SM in 2011, was admitted for planned aortic valve replacement. A preoperative echocardiography revealed severe aortic valve stenosis with a calculated aortic valve area of 0.7 cm^2^, a maximal pressure gradient of 78 mmHg, a mean pressure gradient of 44 mmHg as well as a left ventricular ejection fraction of 60%. No left ventricular hypertrophy was present. A preoperative coronary angiography revealed a single-vessel coronary artery disease with a 30% stenosis of the right coronary artery (RCA). The patient had a medical history of arterial hypertension, obesity, osteoporosis and systemic mastocytosis.

**Figure 1:**
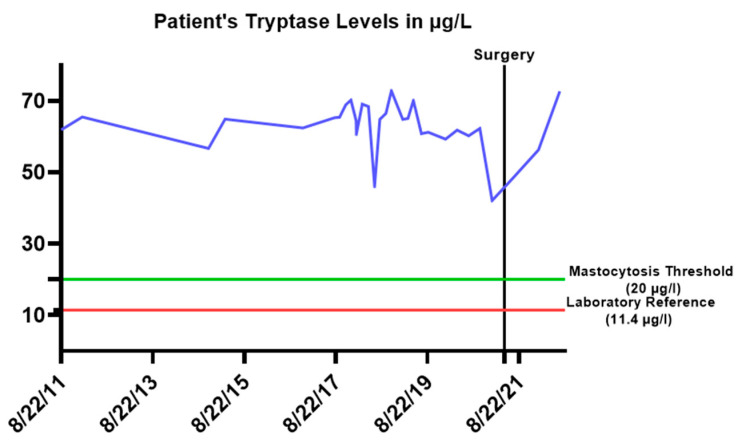
The patient’s tryptase levels were measured from 2011 to 2022 (blue line). As shown, the follow-up levels of the patient were elevated all along the treatment since the first diagnosis date in 2011. The laboratory reference level was 11.4 µg/L.

The disease first manifested as cutaneous mastocytosis with urticaria pigmentosa, associated with high tryptase levels (08.2011: 61.9 µg/L) (Figure 1). A bone marrow biopsy taken in 2011 showed a bone marrow infiltration of approximately 10%. Moreover, c-KIT negativity was determined. Thus, the patient fulfilled the criteria of SM with 1 major (multifocal mast cell aggregation) and 3 minor criteria (Serum tryptase > 20 µg/L, mast cells showed aberrant CD 25 and CD2 and at least 25% of the mast cells were spindle cell/atypical). 

The course of the patient’s SM was mild with predominantly dermatologic symptoms (urticaria pigmentosa), which were successfully treated with the administration of Cetirizine 5 mg once daily and by ultraviolet light therapy treatment (PUVA) in the fall and winter months. Osteoporosis was stable under a regular administration of Zoledronic acid twice yearly; previously, it had been administered every 4 to 6 weeks. As a preventive measure, the patient was provided with an emergency kit containing an adrenaline autoinjector for the emergency treatment of acute allergic reactions with anaphylaxis, Celestamine liquid and Fenistil drops. 

The current preoperative course on the part of SM was uncomplicated. Considering the medical history, a perioperative protocol was established consisting of an intravenous injection of 8 mg of dexamethasone 1 h and 50 min before surgery in order to prevent potential anaphylactic complications. Total intravenous anesthesia was induced and maintained with propofol, esketamine and sufentanil. Cisatracurium was used as a muscle relaxant. However, in this patient, inhalational anesthesia may be preferred according to the literature [7]. 

During the normothermic (35.8 °C) cardiopulmonary bypass, the heavily calcified, stenotic tricuspid aortic valve was replaced with a biological 23 mm Edwards Perimount Magna Ease Aortic valve prosthesis via partial medial sternotomy. The cardiopulmonary bypass time and the cross-clamping time were 134 min and 96 min, respectively. Subsequently, the patient was weaned from cardiopulmonary bypass successfully with moderate doses of catecholamines (dobutamine 0.00625 mg/kg/min and norepinephrine 0.167 µg/kg/min), and the systemic anticoagulation with heparin was reversed by a slow administration of protamine over 26 min, in a 1:1 manner. 

**Figure 2:**
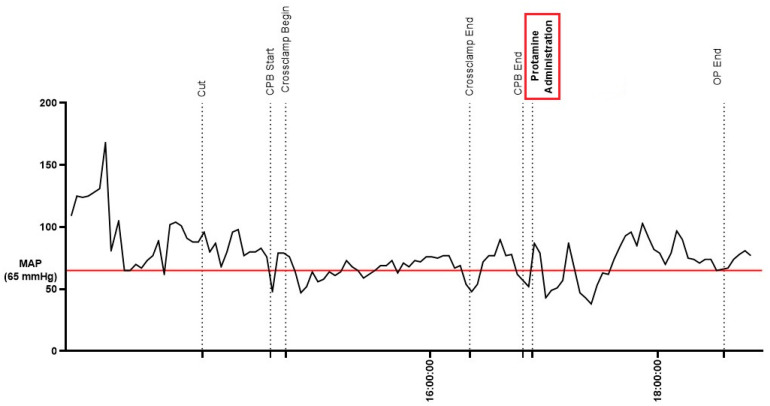
Hemodynamic follow-up during aortic valve replacement illustrating relevant event during surgery. A reference mean arterial pressure (MAP) was traced (red line), with a cutoff value of 65 mmHg.

During protamine administration, the patient deteriorated and developed vasoplegia and a critical hypotension. Assuming that the causative factor was a histamine liberation due to an anaphylactic reaction attributable to the pre-existing systematic mastocytosis, epinephrine, antihistamines and corticosteroids were injected (Figure 2).

The patient could be stabilized and transferred to the intensive care unit under moderate inotropic and vasopressor support. On the first postoperative day, the patient was successfully extubated, and the administration of all catecholamines could be stopped without any further sequelae.

During the patient’s stay in the intensive care unit, she temporarily developed brown maculopapular patches and freckles due to the abnormal accumulation of mast cells in the skin, typical for the cutaneous form of mastocytosis, which had remitted by the time of discharge.

On postoperative day 1 (POD), pronounced superficial skin redness and hemorrhage in the sternal area were noticed. The sternum was stable at all times, and the wound was successfully treated with Cultimed Sylter^®^ dressing. 

When inflammatory parameters and fever occurred, calculated antibiotic therapy was given, under which the symptomatology subsided.

The further hospital stay was free of severe complications with the prophylactic administration of Levocetirizine 5 mg daily, and the patient could be discharged on POD 13 in a stable condition. Allergies to protamine, and isophane insulins (crystalline suspensions of a complex of protamine sulfate and insulin, so-called neutral protamine hagedorn [NPH-]), were described in the discharge letter to prevent re-administration and further critical anaphylactic reactions in this patient.

## 3. Methods

### 3.1. Systematic Review—Search Strategy

The search strategy was conducted in accordance with the PRISMA-S extension of the PRISMA statement for reporting literature searches in systematic reviews. We followed the Preferred Reporting Items for Systematic Reviews and Meta-Analyses (PRISMA) guidelines to conduct this systematic review. 

The following databases were systematically searched: Pubmed, SCOPUS and Web of Science using the search terms “Systemic Mastocytosis” and “cardiac surgery” “heart surgery”, “extracorporal circulation”, “cardiopulmonary bypass”, “heart lung machine” or “HLM” (Tables S1 and S2). 

We included all articles published in English prior to September 2022 (Figure S1). All included articles were identified by two authors (MS and ASK). Afterwards, the full texts of all selected studies were reviewed. Additional articles were manually identified by screening the cited references. No study registries or other online resources were searched. No date, language or study design filters were used. No additional studies were sought by contacting authors. 

Studies were considered eligible for inclusion if they (1) involved individuals with SM undergoing cardiac surgery and (2) were published in English. All studies describing the clinical features or complications of a subject with SM undergoing cardiac surgery were included. Conversely, studies were excluded if they (1) were duplicate publications or (2) did not report patients undergoing cardiac surgery. 

All original searches were conducted on 20 September 2022. 

### 3.2. Study Quality Assessment

The quality of the included studies was evaluated by MS, ASK and FH.

### 3.3. Data Extraction

Data were extracted independently with a standardized form by two authors (MS and ASK). When consensus could not be reached, disagreements were solved by consultation or discussion with a third reviewer. 

The following data were extracted and summarized in Table 1: the first author and year of publication, geographic region, participants, age composition, type of surgery, time of extracorporal circulation (ECC), bypass time, cross-clamping time, body temperature in surgery, tryptase level, method of diagnosis of SM and day of discharge. Moreover, the different premedication strategies, induction and maintenance of anesthesia and postoperative strategies were retrieved and summarized in Table 2.

## 4. Results

In total, 136 unique citations were identified after an initial search. Of these, 109 were excluded after titles and abstracts were screened, mainly because they were duplicates, reviews or unrelated to the current study. Then, the full text of 27 articles was reviewed, 17 of which were excluded. Finally, a total of 11 studies were eligible and included in the systematic review. The characteristics of the 11 included studies were summarized in Table 1. 

The data were published between 1986 and 2022 involving a total of 11 patients. Four (36.4%) studies were conducted in European countries, namely Poland, Belgium and Spain, whereas seven (63.6 %) studies were in non-European countries, namely the USA, Columbia and India. All studies sampled SM patients from the clinic-based population. All patients reported suffered from SM and were treated in a cardiac surgery department. In total, 10 out of the 11 patients received ECC during surgery. Different types of surgery are summarized in Table 1.

Adverse effects have occurred in the cases from Duggal et al. and Ripoll et al. In the case of Duggal et al. episodes of severe hypotension occurred at two different operation instances during induction, where the surgery had to be postponed twice [9]. In the case of Ripoll et al. hypotension occurred during protamine administration [12]. As a result, the patient had to be connected back to the CPB. In the case presented here, hypotension also occurred due to protamine administration. However, re-CPB could be avoided by the prompt treatment through the administration of epinephrine, antihistaminic and corticosteroids (Table 1).

## 5. Discussion

Systemic mastocytosis (SM) can induce dangerous anaphylactic reactions during cardiac surgery. Therefore, this rare disease is of importance for both cardiac surgeons and cardiac-anaesthesiologists.

Because there are scarce data on the optimal intra- and perioperative care of patients with SM undergoing cardiac surgery, we present the complicated intraoperative course of an adult woman with SM who required aortic valve replacement for severe aortic valve stenosis. This case report highlights the importance of careful preoperative planning and coordination between cardiac surgeons and anesthesiologists, particularly in vulnerable patients with a rare medical condition such as SM. 

SM is a non-hereditary disease with a nearly equal gender distribution [18]. The estimated European prevalence is between 1:7700 and 1:10,400 [19], but accurate data on the incidence and prevalence in various regions and countries worldwide are lacking [20]. 

Since 2016, SM has been a separate category in the WHO classification with five subtypes, including indolent SM (ISM), smoldering SM (SSM), aggressive SM (ASM), SM with an associated hematological neoplasm (SM-AHN) and the mast cell leukemia (MCL), which is no longer classified as a myeloproliferative disease [21]. Mastocytosis can occur not only as a systemic but also as a cutaneous form, referred to as urticaria pigmentosa [13]. However, one major criterion with a minor criterion or 3 minor criteria are necessary for the establishment of the diagnosis of systemic mastocytosis (Table 3).

SM is not curable, and there are only preventive or limited treatment options, including symptomatic care and supportive measures, such as trigger-avoidance, pharmacotherapy with antihistamines (H1/H2), antileukotriene drugs, cromoglicic acid and Vitamin C. In advanced SM, therapy is indicated with tyrosine-kinase inhibitors (e.g., midostaurin, avapritinib) or cytoreductive therapy (e.g., cladribine, hydroxyurea) for mast cell debulking. In severe cases, such as mast cell leukemia, allogenic stem cell transplantation can be a last option [3,23]. 

In SM, the degranulation of the neoplastic mast cells that accumulate in various organs may be triggered by chemical, physical or psychological factors (e.g., alcohol, physical effort and high or low temperatures, stress, emotions); infections; or medical procedures, including sedation, analgesia or multidrug pharmacotherapy [5,6,13]. 

The excess release of messenger substances, such as histamine, leukotrienes, prostaglandins, platelet-activating factor, interleukins, proteases, tumor necrosis factor and cytokines, can cause a severe allergic reaction, anaphylactic shock, severe inflammation, significant cardiovascular alterations and organ damage (Table 4) [4,23].

Certain medications, such as acetylsalicylic acid, amphotericin B, quinine, sedatives and analgesics (lidocaine, tetracaine, procaine, morphine, codeine, etomidate, thiopental, succinylcholine, enflurane, isoflurane), or iodine-based contrast media are also potential trigger factors [5,6,13]. However, many proposals of contraindicated and “safe” medications are contradictory and not supported by scientific evidence. Based on their experience, previous case reports and the recommendations from allergy, immunology and hematology departments, Duggal et al. proposed recommendations for perioperative management (Table 5) [9].

To prevent or attenuate anaphylactic reactions, several prophylactic protocols have been proposed, including the administration of histamine-1/histamine-2 blockers, corticosteroids, Cromoglicic acid and benzodiazepines, but none of them has shown any real advantage [1,9,11].

In acute hypotension, the drug of choice is epinephrine, which has not only inotropic, chronotropic and vasopressor effects, but may also prevent further mast cell degranulation [9].

There is no doubt that life-threatening complications can occur intraoperatively and perioperatively in patients with SM, particularly during major surgical procedures. 

During cardiac surgery with CPB, contact of the blood components with the artificial surface of the bypass circuit, clamping and declamping of the aorta, ischemia-reperfusion injury, endotoxemia and surgical trauma may lead to the uninhibited release of mast cell mediators and consecutively severe allergic reactions [4]. Therefore, preoperative preventive treatment and extremely careful intraoperative monitoring are mandatory. 

As the present case shows, such an anaphylactic reaction can develop at any time during or after the operation. In individual cases, it will not be possible to prove which particular factor has triggered the allergic reaction. In the present situation, however, the immediate temporal relationship with the administration of protamine to abolish anticoagulation from unfractionated heparin suggests that this substance caused the anaphylaxis.

In SM, it is quite conceivable that adverse reactions to protamine, such as severe hypotension, could be mediated by the release of inflammatory mediators, including histamine [25]. Therefore, as a precautionary measure, it should be considered to restrict the use of protamine in SM patients. 

Alternatives for the reversion of anticoagulation after CPB could exist. Indeed, prothrombin complex concentrate (PCC) and fresh-frozen plasma (FFP) have been investigated [26]. They have found that PCC is more helpful than FFP for this purpose. However, the small number of patients in their study did not support its safe use. The other alternative is the recombinant activated factor VII. Ponschab et al. have described the use of recombinant activated factor VII in patients undergoing cardiac surgery [27]. They have described a significant increase in stroke in their patient population. Thus, the risk of postoperative hemorrhaging and the increased risk of thromboembolic events are of the utmost importance. Thus, in our institution, the reversal of heparin with protamine is still the standard procedure in patients with SM, as no other method for on-pump surgery has been validated. 

In summary, all patients with SM undergoing medical procedures or even cardiac or non-cardiac surgery should be considered as high-risk cases for mast cell degranulation, increased histamine levels and anaphylactic reactions. All potential causative treatments or drugs should be avoided or administered slowly and with a reduced dose, if possible, to avoid hypersensitivity reactions (Table 6). In addition, preoperative management and treatment plans should be established to avoid critical situations that may be responsible for mast cell degranulation, and to minimize the effect of released mediators [11].

Acute degranulation treatment is suggested following recent guidelines for the treatment of anaphylaxis [28]. 

## 6. Conclusions

In vulnerable patients with a rare disease such as SM, careful preoperative preparation and cooperation between cardiac surgeons, anesthesiologists and hemato-oncologists are imperative to successfully manage a life-threatening intraoperative complication. The time window, in our case, was very narrow, and only such close cooperation made it possible to provide successful treatment to the patient. 

As can be seen from the present case report, SM is a rare but serious condition. This is especially true when cardiac surgery becomes necessary. The data available in the literature to date are sparse but provide detailed guidance for appropriate preventive measures and therapeutic steps that may become necessary. With the limited data available, it is necessary to expand clinical research in SM.

## 7. Strength and Limitations 

There are several limitations in our study. First, the major limitations of this study result from the quality of reporting in the included studies. Second, patients derived from different geographic regions and ethnic backgrounds had different types of cardiac surgery. Furthermore, significant unassessed confounding factors may have influenced the observed course. Lastly, this systematic review included studies published in English, so added research in other languages is needed to confirm the findings.

In contrast, our study also has strengths. We included the latest literature, which means that the findings are applicable to present practices. Our data will provide clinicians with some helpful guidance in the management of patients with SM undergoing cardiac surgery. All these points highlight the need for high-quality, collaborative registries. 

## Figures and Tables

**Table 1:** Clinical characteristics of published cases.

Report	Age (Years) Sex	Surgical Approach	CPB		Body Temperature	Tryptase Level (µg/L)	Comments	Diagnosis of SM	Discharge POD
			BT (min)	CCT (min)					
1. Wanamaker, K et al., 2012 [4]	72, F	AVR	81	62	ND	ND		Diagnosed in 1982 by elevated urinary NT-methylhistamine and NT-methylimidazole acetic acid (2× baseline)	6
2. Damodar, S et al., 2006 [8]	14 years, F	ASD Closure	ND	ND	ND	3.36	Uneventful OP	BM + splenic biopsy, IHC (CD117), impression (benign lesion with eosinophilia and MC infiltrate)	14
3. Duggal, N et al., 2015 [9]	53, M	TAAR	101	73	Hypothermia (32 °C)	154	During anesthesia induction, episodes of severe hypotension: surgery needed to be postponed twice	ND	4
4. Sukrithan, V et al., 2015 [10]	59, M	Pericardial window	NA	NA	NA/ND	115/154		c-Kit + in pericardial tissue, CD25+ MC in pericardial infiltrate, blood PCR: + D816V	
5. Giraldo-Grueso, M et al., 2018 [11]	63, F	MVR	94	78	Normothermia	11.8		In BM biopsy	
6. Ripoll, J et al., 2019 [12]	69, M	LAD Unroofing	ND	ND	ND	143	After protamine administration: re-CBP due to severe hypotension, re-exploration due to bleeding	+D816V; 5% burden of MC	9
7. Rozewicz-Juraszek, M et al., 2017 [13]	50, F	AVR	ND	ND	ND	ND	ND	ND	8
8. Beckers, S et al., 2018 [14]	57, M	ACB	77	23	Normothermia	76	ND	+D816V	10
9. Moro, J et al., 2008 [15]	56, F	HTx	ND	ND	ND	173	ND	ND	ND
10. Martín Serrano, P et al., 2019 [16]	71, M	AVR + ACB	59	49	Normothermia	61.4	ND	+D816V; 2% MC	6
11. Trubov, N et al., 1986 [17]	51, M	ACB	140	4 × 10-12	Hypothermia (25 °C)	ND	ND	5+ mast cells in an HPF	ND
12. Suleiman MN et al., 2023	66, F	AVR	134	96	Normothermia	61.9		10% spindle cellular mast cell infiltration of the BM; aberrant expression of CD25 and CD2	13

ACB: Aortocoronary Bypass; ASD: Atrial Septal Defect; AVR: Aortic Valve Replacement; BM: Bone Marrow; BT: Bypass Time; CCT: Cross-Clamp Time; CPB: Cardiopulmonary Bypass; +D816V: positive for mutation of Aspartate by Valine in position 816; F: female; HPF: High-Power Field; HTx: Heart Transplant; IHC: Immunohistochemistry; LAD: Left Anterior Descending; M: male; MC: Mast Cell; MVR: Mitral Valve Replacement; NA: Non-Applicable; ND: Non-Disclosed; OP: Operation; TAAR: Thoracic Aortic Aneurysm Repair.

**Table 2:** Different anesthesia protocols, pre- and postoperative therapies used to control SM.

Report	Preoperative Management	Induction of Anesthesia	Maintenance of Anesthesia	Postoperative Management
1. Wanamaker, K et al., 2012 [4]	24 h before the surgery: - Methylprednisolone 100 mg IV every 8 h 1 h before induction: - Diphenhydramine 50 mg IV- Famotidine 20 mg IV - Solu-medrol 125 mg IV	- Etomidate - Isoflurane - Pancuronium	- Isoflurane - Fentanyl	Until discharge: - 5-day steroids tapering - Scheduled diphenhydramine -Ranitidine
2. Damodar, S et al., 2006 [8]	- Methylprednisolone 750 mg IV bolus - Injection hydrocortisone 100 mg IV - 6 h before surgery: Ranitidine and cetirizine	- Fentanyl - Midazolam - Pancuronium		For 2 weeks: - Steroids - Ranitidine - Cetirizine
3. Duggal, N et al., 2015 [9]	24 h before surgery: - Prednisone 50 mg PO 12 h before surgery: - Prednisone 50 mg PO - Diphenhydramine 25–50 mg PO/IV - Ranitidine 150 mg PO or 50 mg IV - Montelukast 10 mg PO 2 h preoperative: - Prednisone 50 mg PO - Diphenhydramine 25–50 mg PO/IV - Ranitidine 150 mg PO or 50 mg IV - Montelukast 10 mg PO	OP 1 - Midazolam - Fentanyl - Lidocaine - Propofol - Vecuronium OP 2 and 3 - Propofol - Fentanyl - Lidocaine - Succinylcholine	OP 3 - Inhaled Isoflurane - Dexmedetomidine - Fentanyl	- Montelukast 10 mg PO - Ranitidine 150 mg PO - Prednisone taper over 5 days - Cetirizine 10 mg PO
4. Sukrithan, V et al., 2015 [10]	ND	ND	ND	
5. Giraldo-Grueso, M et al., 2018 [11]	- Cetirizine 10 mg daily - Ketotifen 1 spoon daily - Ranitidine 10 mg daily 24 h before the surgery: - Prednisone 50 mg Q8H - Lorazepam 2 mg every Q12H 1 h before induction: - Dexchlorpheniramine 5 mg IV - Ranitidine 100 mg IV - Methylprednisolone 125 mg IV	- Fentanyl - Midazolam - Etomidate - Vecuronium	- Isoflurane	- Cetirizine 10 mg daily - Methylprednisolone 40 mg IV for 5 days
6. Ripoll, J et al., 2019 [12]	ND	ND	ND	For reversal of Heparin, intra-OP lower dose of protamine with: - Dexamethasone - Diphenhydramine
7. Rozewicz-Juraszek, M et al., 2017 [13]	13 h before surgery: - Dexamethasone 8 mg IV 7 h before surgery: - Dexamethasone 8 mg IV - Cetirizine 10 mg IV - Ranitidine 150 mg IV 1 h before surgery: - Dexamethasone 8 mg IV	- Propofol - Fentanyl	ND	- Dexamethasone 8 mg IV - Cetirizine 10 mg IV - Ranitidine 150 mg IV
8. Beckers, S et al., 2018 [14]	24 h before surgery: - Prednisolone 50 mg PO - Levocetirizine 10 mg PO 12 h before surgery: - Prednisolone 50 mg PO - Levocetirizine 10 mg PO - Ranitidine 150 mg PO - Montelukast 10 mg PO 2 h before surgery: - Prednisolone 50 mg PO - Levocetirizine 10 mg PO - Ranitidine 150 mg PO - Montelukast 10 mg PO -Lercanidipine 10 mg PO 1 h before surgery: - Promethazine HCl 50 mg IM	- Midazolam 3 mg IV - Propofol 250 mg IV - Sufentanil 20 µg IV - Cisatracurium 14 mg IV	- Sevoflurane - Sufentanil Infusion	- Cetirizin 10 mg IV - Ranitidine 150 mg IV - Montelukast 10 mg IV - Hydrocortisone 40 mg IV
9. Moro, J et al., 2008 [15]	4 h before surgery: - Prednisone 90 mg IV 1 h before induction: - Prednisone 90 mg IV - Dexchlorpheniramine 5 mg IV - Ranitidine 50 mg IV 30 min before induction: - Diazepam 10 mg PO	- Etomidate - Vecuronium - Fentanyl	- Sevoflurane	-Mycophenolate mofetil 1g every 12 h - Cyclosporine (level between 200 and 300 ng/mL) - Deflazacort
10. Martín Serrano, P et al., 2019 [16]	24 h before surgery: - Montelukast 10 mg PO - Methylprednisolone 125 mg IV - Lorazepam 1mg SL 1 h before surgery: - Montelukast 10 mg PO - Methylprednisolone 125 mg IV - Dexchlorpheniramine 5 mg IV - Ranitidine 150 mg IV - Lorazepam 1 mg SL	- Midazolam - Remifentanil IV - Sevoflurane 8% - Rocuronium 100 mg IV	- Sevoflurane 2% - Remifentanil - Rocuronium	ND
11. Trubov, N et al., 1986 [17]	24 h before the surgery: - Chlorpheniramine 9 mg PO - Diphenhydramine 25 mg PO Morning of surgery: - Cimetidine 300 mg PO - Diphenhydramine 50 mg IM - Methylprednisolone 100 mg IM	ND	ND	ND
12. Suleiman MN et al., 2023	2 h preoperatively: - Dexamethasone 8 mg IV	- Propofol - Sufentanil - Cisatracurium	- Propofol - Sufentanil - Cisatracurium	- Levocetirizine 5 mg

h: Hour(s); IM: Intramuscular; IV: Intravenous; min: Minutes; PO: Per Os; SL: Sublingual.

**Table 3:** Major and minor criteria for the fulfillment of SM diagnosis (according to Valent P et al. [22]).

Criteria	
Major	Multifocal dense infiltrates of MCs (≥15 MCs in aggregates) in BM biopsies and/or in sections of other extracutaneous organ(s)
Minor	1. >25% of all MCs are atypical cells (type I or type II) on BM smears or are spindle-shaped in MC infiltrates detected on sections of visceral organs
	2. *KIT* point mutation at codon 816 in the BM or another extracutaneous organ
	3. MCs in BM or blood or another extracutaneous organ exhibit CD2 and/or CD25
	4. Baseline serum tryptase level >20 ng/mL (in case of an unrelated myeloid neoplasm, item 4 is not valid as an SM criterion)

**Table 4:** Mast cell mediator and accompanying symptoms (according to Konrad et al. [24]).

Symptoms	Mediator
Vasodilation/hypotension	Histamine Prostaglandin D2
Fatigue/cachexia/weight loss	TNF-α
Fever	IL-6
Urticaria	Histamine Prostaglandin D2
Abdominal pain	Histamine
Diarrhea	Histamine
Osteoporosis/osteopenia	IL-6 Tryptase
CNS syndromes	Histamine Prostaglandin D2

**Table 5:** Recommendation for the pharmacological prevention of mast cell degranulation in SM according to Duggal et al. modified [9].

24 h Preoperative	12 h Preoperative	2 h Preoperative	Postoperative Care
Prednisone 50 mg PO	Prednisone 50 mg PO Diphenhydramine 25–50 mg PO/IV Famotidin 40 mg PO Montelukast 10 mg PO	Prednisone 50 mg PO Diphenhydramine 25–50 mg PO/IV Ranitidine 150 mg PO or 50 mg IV Montelukast 10 mg PO	Prednisone taper over 5 days (40 mg–30 mg–20 mg–10 mg–5 mg–Off) Continue Montelukast 10 mg PO daily Continue Ranitidine 150 PO daily Avoidance of direct mast cell activators such as opiates (morphine, codeine) Start Cetirizine 10 mg PO daily (at discretion of primary allergist)

**Table 6:** Overview of potential triggers of mast cell degranulation and how to prevent them.

Potential Trigger Factors for Mast Cell Degranulation *	Avoid	Prefer
In general	Chemical factors, physical effort, high- or low-temperature psychological factors (anxiety, stress, emotions), alcohol, infections	
General medical procedures	Sedation, analgesia, multidrug pharmacotherapy	Good pain control: novel local anesthesia blockage and acetaminophen. ESP block [11]
Certain medications	Acetylsalicylic acid, amphotericin B, quinine, sedatives, and analgesics (lidocaine, tetracaine, procaine, morphine, codeine, etomidate, thiopental, succinylcholine, enflurane, isoflurane), iodine-based contrast media Betablockers [13] ACE-Inhibitors [13]	
Anesthetic drugs	Mivacurium, atracurium, codeine, morphine	Fentanyl and related medications such as remifentanil, sufentanil and alfentanil can be used with relative safety [11]
Cardiac surgery	Cardiopulmonary bypass (CPB), hypothermia, contact of blood components with artificial surface of the bypass circuit, clamping of the aorta, ischemia-reperfusion injury, endotoxemia, surgical trauma, protamine	Prefer normothermia [11]

* without Claim to Completeness.

## Data Availability

Not applicable.

## Supplementary Materials

The following supporting information can be downloaded at: https://www.mdpi.com/article/10.3390/jcm12031156/s1, Figure S1: PRISMA 2020 Flow Diagram; Table S1: PRISMA 2020 Checklist; Table S2: Search Strategy. Reference [29] is cited in the supplementary materials.
