# Supplementary material for: A Practical Approach to Systemic Mastocytosis Complications in Cardiac Surgery: A Case Report and Systematic Review of the Literature
Source: J Clin Med. 2023 Feb 1;12(3):1156. doi: 10.3390/jcm12031156 (PMC9917416; doi:10.3390/jcm12031156)
Supplement: Supplementary file 1 [file jcm-12-01156-s001.zip › Figure S1. 02_PRISMA_2020_flow_diagram_new_SRs_v2.pdf]

PRISMA 2020 flow diagram for new systematic reviews which included searches of databases, registers and other sources

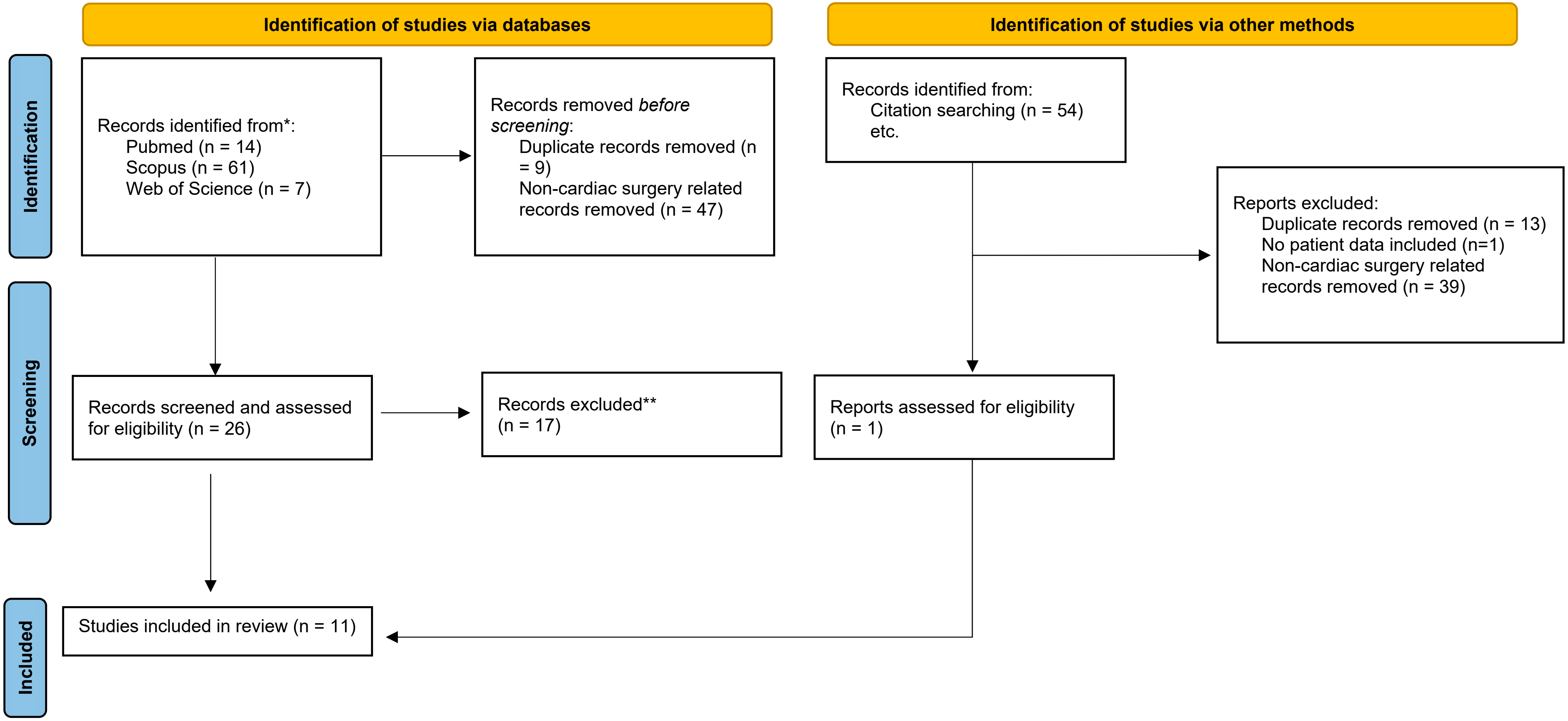

*Consider, if feasible to do so, reporting the number of records identified from each database or register searched (rather than the total number across all databases/registers).
**If automation tools were used, indicate how many records were excluded by a human and how many were excluded by automation tools.

From: Page MJ, McKenzie JE, Bossuyt PM, Boutron I, Hoffmann TC, Mulrow CD, et al. The PRISMA 2020 statement: an updated guideline for reporting systematic reviews. BMJ 2021;372:n71. doi: 10.1136/bmj.n71. For more information, visit: http://www.prisma-statement.org/
