# Supplementary material for: A Practical Approach to Systemic Mastocytosis Complications in Cardiac Surgery: A Case Report and Systematic Review of the Literature
Source: J Clin Med. 2023 Feb 1;12(3):1156. doi: 10.3390/jcm12031156 (PMC9917416; doi:10.3390/jcm12031156)
Supplement: Supplementary file 1 [file jcm-12-01156-s001.zip › Table S2. 02_Search Strategy Table.pdf]

Supplementary Table S2 – Search Strategy

Database	Search strategy	References identified
**Pubmed**	((systemic mastocytosis) AND ((cardiac surgery) OR (Extracorporal circulation) OR (cardiopulmonary bypass) OR (heart lung machine) OR (HLM))	14
**SCOPUS**	((TITLE-ABS-KEY (systemic mastocytosis)) AND ((cardiac surgery) OR (Extracorporal circulation) OR (cardiopulmonary bypass) OR (heart lung machine) OR (HLM)))	61
**Web of Science**	TOPIC: ((systemic mastocytosis) AND (cardiac surgery)))	7
